# YeastWeb: a workset-centric web resource for gene family analysis in yeast

**DOI:** 10.1186/1471-2164-11-429

**Published:** 2010-07-13

**Authors:** Yanhui Chu, Xiaohuan Yuan, Yanqin Guo, Yufei Zhang, Yan Wu, Haifeng Liu, Dan Wu, Haihua Bao, Lixin Guan, Xiudong Jin

**Affiliations:** 1 Heilongjiang Key Laboratory of Anti-fibrosis Biotherapy, Mudanjiang Medical University, Heilongjiang 157011, China; 2 Affiliated Hospital of Mudanjiang Medical University, Mudanjiang Medical University, Heilongjiang 157011, China

## Abstract

**Background:**

Currently, a number of yeast genomes with different physiological features have been sequenced and annotated, which provides invaluable information to investigate yeast genetics, evolutionary mechanism, structure and function of gene families.

**Description:**

YeastWeb is a novel database created to provide access to gene families derived from the available yeast genomes by assigning the genes into putative families. It has many useful features that complement existing databases, such as SGD, CYGD and Génolevures: 1) Detailed computational annotation was conducted with each entry with InterProScan, EMBOSS and functional/pathway databases, such as GO, COG and KEGG; 2) A well established user-friendly environment was created to allow users to retrieve the annotated genes and gene families using functional classification browser, keyword search or similarity-based search; 3) Workset offers users many powerful functions to manage the retrieved data efficiently, associate the individual items easily and save the intermediate results conveniently; 4) A series of comparative genomics and molecular evolution analysis tools are neatly implemented to allow users to view multiple sequence alignments and phylogenetic tree of gene families. At present, YeastWeb holds the gene families clustered from various MCL inflation values from a total of 13 available yeast genomes.

**Conclusions:**

Given the great interest in yeast research, YeastWeb has the potential to become a useful resource for the scientific community of yeast biologists and related researchers investigating the evolutionary relationship of yeast gene families. YeastWeb is available at http://centre.bioinformatics.zj.cn/Yeast/.

## Background

Yeast biology studies have been greatly accelerated since the sequencing of the first yeast genome *Saccharomyces cerevisiae*. Undoubtedly, the availability of a complete yeast genome provides invaluable information to investigate yeast genetics, evolutionary mechanism, structure and function of gene families. As a consequence, publicly available comprehensive bioinformatics databases and tools need to be established for retrieving various genome-level sequence data to facilitate highly rapid progress in yeast biology research. First and foremost in the yeast community, for example, the *Saccharomyces *Genome Database (SGD) and the MIPS Comprehensive Yeast Genome Database (CYGD) are public resources with integrated genetic, genomic, and functional data of the budding yeast *S. cerevisiae *[1,2]. The Comparative Fungal Genomics Platform (CFGP) was developed to allow access fungal genome sequences and support comparative genomics analyses [3]. Génolevures is a online database, which allow users to perform comparative genomics and molecular evolution investigation of hemiascomycetous yeasts [4].

Currently, the emergence of high-throughput sequencing technologies has remarkably sped up whole-genome de novo sequencing in a rapid and cost-effective fashion. At present, there are 13 yeast genomes with different physiological features available on the KEGG database [5]. Such genome data resources have provided us a golden opportunity to investigate the yeast genes and gene families and further their evolutionary and functional implication. In this study, a well-organized database YeastWeb is specifically constructed to provide various detailed information of genes and gene families from the available yeast genomes. It has many useful features that complement existing databases, such as SGD, CYGD and Génolevures. Each gene and gene family entry is extensively annotated by scanning through InterProScan, EMBOSS and functional/pathway databases such as COG, KEGG and Gene Ontology. Through the design of workset, all of the retrieved data is well integrated and the intermediate work result can be easily saved for future use. In addition, YeastWeb can help researchers to elucidate the evolutionary relationships of yeast gene families and to carry out in-depth comparative sequences analyses. Given the great interest in yeast research, YeastWeb has the potential to become a useful resource available for the scientific community of yeast biologists and related researchers.

## Construction and content

### Gene family assignment

The available 13 yeast genome sequences were obtained from the KEGG database [5]. These genomes are *S. cerevisiae *(5,880 genes), *S. bayanus *(9,344 genes), *K. waltii *(5,213 genes), *V. polyspora *(5,336 genes), *Y. lipolytica *(6,472 genes), *S. paradoxus *(8,908 genes), *A. gossypii *(4,725 genes), *D. hansenii *(6,324 genes), *C. albicans *(6,317 genes), *S. mikatae *(8,972 genes), *K. lactis *(5,335 genes), *P. stipitis *(5,816 genes) and *C. glabrata *(5,191 genes). To assign the proteins into families, an all-against-all BLAST search was conducted for all the predicted proteins from the 13 yeast genomes using the BLASTP program (-e = 10^-5^, -b = 10,000, -v = 10,000). Then, the protein families were generated using the TribeMCL program [6]. The TribeMCL program is proved to be rather accurate since it considered the multidomains, fragments of proteins and promiscuous domains during the clustering process [7]. Under different inflation values of 1.5, 2.5, 3.0, 4.0 and 5.0, a total number of 83,833 proteins derived from all the 13 yeast genomes were clustered into 18,275, 22,698, 23,839, 25,422 and 26,457 families, respectively. All these sequence family data is available for free download without any restriction. Furthermore, the download page provides the utilities to batch download nucleic acid and/or amino acid sequences according to MCL inflation value, cluster size and species.

### Database construction

YeastWeb is designed as a relational database and hosted on an Apache HTTP server running on Linux operating system. YeastWeb web interface is implemented in an operating-system independent way and has been tested to work well in Internet Explorer 6.0, Firefox 3.0.1, and Opera 10.00 browsers. All the annotations for the genes and genes families are organized into various separate MySQL database tables, which can be retrieved by the Structure Query Language (SQL) easily. PHP is used to connect the database and produce user-friendly HTML front-end queries dynamically.

## Utility

### Workset configuration and data retrieval

**Figure 1:**
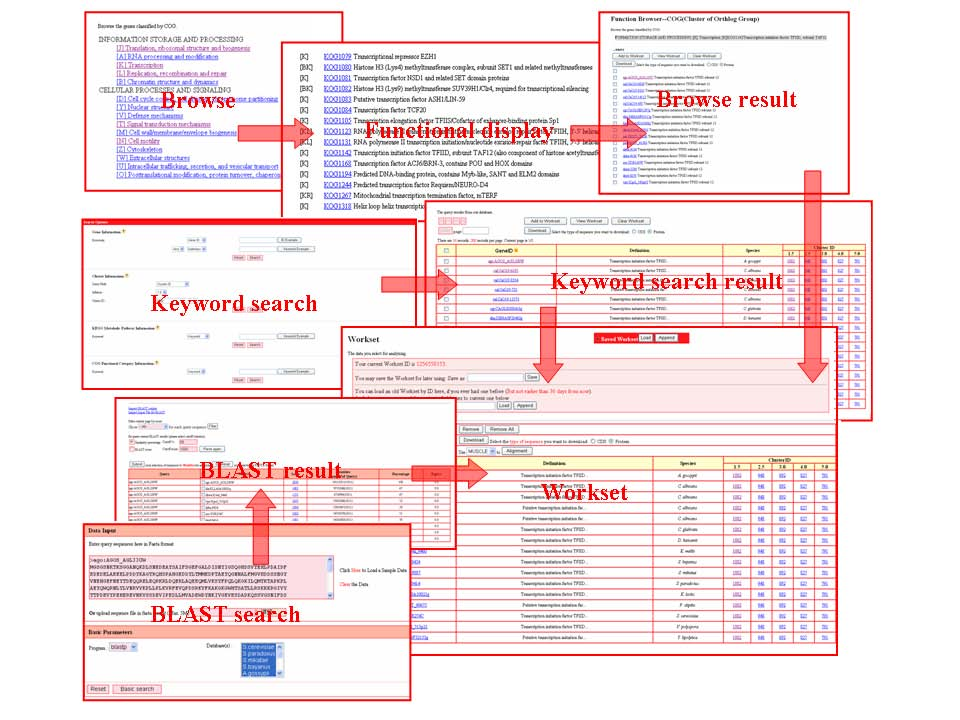
**Screenshots of data retrieval and workset configuration**. Users can base on functional classification browser, keyword search or similarity-based search to retrieve yeast gene families. All the retrieved results can be added into the workset for

A key feature of YeastWeb is that its web interface has been well organized and presented in the form of workset (Fig. 1). Through the workset, all the data sets in the YeastWeb can be easily linked together and the intermediate work result can be saved. For the data stored in the workset, a succession of analyses can be achieved using a number of implemented comparative genomics and molecular evolution analysis tools. Prior to construction of a personalized workset, genes or gene families could be first retrieved using functional classification browsers, keyword searches or BLAST searches.

The browse function allows users to visualize gene families that are in the same COG functional category or KEGG metabolic pathway (Fig. 1). COG and KEGG function categories assignment of a given category in YeastWeb is pre-computed using the BLAST program with an E-value of 1e^-5^. Once users click a given COG category or KEGG metabolic pathway (organized hierarchically), a list of genes belonging to such category will be presented. Then, users can select specific or all of the retrieved items and add them to the workset.

The search function is available to allow users to access the genes and gene families by keyword. Four searching options are available in the search page: gene information, cluster information, KEGG metabolic pathway information and COG functional category information. For gene information, users can retrieve genes of interest based on their gene ID and function description. For cluster information, users can access the interested gene families based on cluster ID, cluster size and cluster description associated with different MCL inflation values. For the KEGG metabolic pathway and COG functional category information, user can retrieve interest genes based on pathway/COG ID or description. The retrieved item shows classification information, including gene ID, function definition, species and cluster ID under different inflation values. Genes, gene families and related information retrieved from all of these ways can be added into the workset by clicking the "Add to workset" button.

The BLAST tool implemented in YeastWeb works as a useful way to enable the users to search their target sequences or identify homologs in the database. Users can select one or more yeast genomes to perform the sequence similarity searching with the protein databases (BLASTP or BLASTX, depending on the query sequence) or nucleotide databases (BLASTN and TBLASTX or TBLASTN). In addition, a number of advanced parameters (such as E-value, matrix and species) were also offered to perform more specific BLAST searches. The BLAST search results can be visualized in standard BLAST output format. However, especially, the results can be easily parsed and navigated according to specific cut-off of E-value or score set by users with the implement of the ViroBLAST program [8]. All the selected items can be directly saved into workset by click the "Submit" button.

### Workset manipulation and entry description

**Figure 2:**
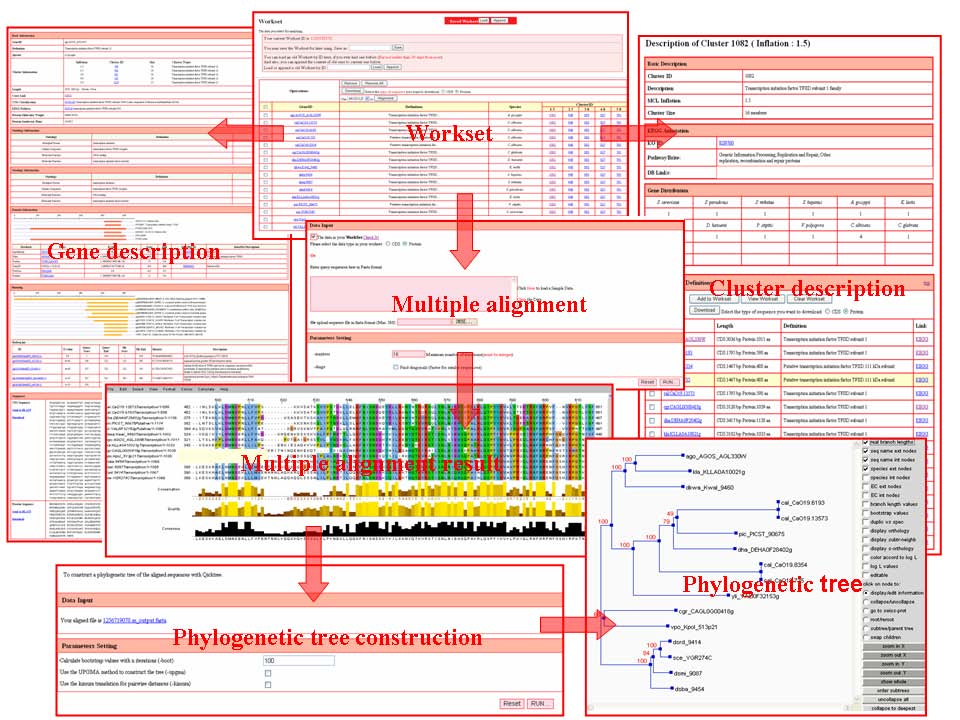
**Screenshots of the gene and gene family description, as well as comparative genomics and molecular evolution analysis**. Clicking specific item in the workset, gene and cluster description can be shown easily. For the data stored in the workset, multiple sequence alignment and phylogenetic tree construction can be achieved used the implemented comparative genomics and molecular evolution analysis tools.

The workset of YeastWeb works like the Microsoft Excel, which offers users a numerous number of powerful functions to manipulate the whole workset table and individual table items (Fig. 2). It allows users to load an existing workset, create a new one, integrate previous records or save current data as designated workset. Each workset is achieved by a unique ID generated by YeastWeb automatically or assigned by a user's own favorite name if users requested. If a user's web browser supports cookies, previous saved workset can be also easily listed. It is noteworthy that, to avoid overwhelming burden of hard disk space, all of user saved workset in YeastWeb are only saved for 30 days. For the items listed in specific workset, users can selectively download, add and remove the items one by one or in bulk.

Detailed gene information includes: 1) Basic information, such as gene ID, function description, species belonging to and cluster ID under different inflation values, sequence length, molecular weight, isoelectric point, COG functional classification and associated KEGG pathway; 2) Gene Ontology information, such as Gene Ontology level, term definition and Gene Ontology ID; 3) Domain organization information, such as domain description from Pfam, SUPERFAMILY, SMART, TIGRFAMs, Gene3 D and Panther et al. 5) Homolog information, such as pre-computed lists of homologs to the entry of Refseq, Swiss-Prot, PDB, KEGG and UniProt (Top five best hits are listed); 6) Sequence information, such as nucleotide and protein sequence. Detailed gene family information also includes: 1) Basic description, such as cluster ID, cluster size, cluster description and MCL inflation value; 2) Family member distribution, which shows the number of genes from different yeast genomes; 3) Individual gene list with gene ID, sequence length and function definition been shown.

### Gene family analysis based on workset

Once a personalized workset have been configured, users can apply the implemented comparative genomics and molecular evolution tools to conduct a succession of analyses to investigate the evolutionary relationship of specific yeast gene family (Fig. 2). Multiple sequence alignment can be easily performed using the "alignment" function, which provides two widely adopted multiple sequence alignment program MUSCLE [9] and ClustalW [10] with a number of advanced parameters. Both protein sequences and nucleotide sequences are allowed to be used to perform the analysis. The visualization of web-based multiple sequence alignment results was implemented using the Jalview program [11], which provide a batch of tools to handle the alignment results and display the sequence features in color using the customized color schemes. However, it should be noted that Jalview is a Java applet program, thus, users have to confirm that a Java Runtime Environment (JRE) is installed on their local machine before visualizing the alignment results. Once a multiple sequence alignment job is finished, the QuickTree program can be directly applied to construct a phylogenetic tree using the neighbor-joining algorithm [12]. The reliability of the tree can be evaluated with the bootstrap method with replications (default value 100). The constructed phylogenetic tree is shown in the user's browser with the implement of ATV program [13].

## Discussion and Conclusions

In comparison with SGD, CYGD and Génolevures, YeastWeb [14] allows all of the genes and gene families derived from the yeast genomes to be accessed via a user-friendly web interface. The web interface is well organized and designed in the form of workset, which is very helpful for users to save their intermediate results. For the data stored in the workset, YeastWeb implemented a number of comparative genomics and molecular evolution analysis tools to allow users to view the multiple sequence alignment and phylogenetic tree of a given gene family. Given the great interests in yeast research, YeastWeb will be a valuable resource available for the scientific community of yeast biologists and related researchers to investigate the evolutionary relationship of yeast gene families. Currently, YeastWeb contains clustered gene families from 13 available yeast genomes and is expected to grow with the rapid development of genome sequencing projects quickly. As more yeast genomes being fully sequenced, YeastWeb will be expanded and updated accordingly.

## Availability and requirements

Project name: YeastWeb: a Workset-centric Web Resource for Gene Family Analysis in Yeast

Project home page: http://centre.bioinformatics.zj.cn/Yeast/

Operating system(s)

For user: Standard WWW browser, such as Firefox3.0,

Internet Explorer 6.0, Firefox 3.0.1, and Opera 10.00 browsers

For server: Linux

Programming language: PHP, MySQL, Perl and BioPerl

License: GNU GPL

Any restrictions to use by non-academics: None

## Authors' contributions

YC and XY analyzed the data and developed website. YC wrote manuscript. YG, YZ and YW took part in the development of database and website. HL, DW, HB helped write manuscript, maintained the database and provided considerable comments on the website construction and manuscript organization. LG and XJ designed and supervised the study. All authors read and approved the final manuscript.
